# The Low-Field Microwave Absorption in EMR Spectra for Ni_50−x_Co_x_Mn_35.5_In_14.5_ Ribbons

**DOI:** 10.3390/ma15176016

**Published:** 2022-08-31

**Authors:** Łukasz Dubiel, Ireneusz Stefaniuk, Andrzej Wal

**Affiliations:** 1 Department of Physics and Medical Engineering, Faculty of Mathematics and Applied Physics, Rzeszow University of Technology, Powstancow Warszawy 12, 35-959 Rzeszow, Poland; 2 Institute of Materials Enginering, College of Natural Sciences, University of Rzeszow, Rejtana 16a, 35-310 Rzeszow, Poland; 3 Institute of Physics, College of Natural Sciences, University of Rzeszow, Pigonia 1, 35-959 Rzeszow, Poland

**Keywords:** EMR, LFMA, magnetic materials, Heusler alloys

## Abstract

This paper contains a detailed study of low-field microwave absorption, which is observed in EMR spectra registered for a series of ${\mathrm{Ni}}_{50-x}{\mathrm{Co}}_{x}{\mathrm{Mn}}_{35.5}{\mathrm{In}}_{14.5}$ (x=0,3,5) Heusler alloys polycrystalline in situ and annealed at 1173 K ribbons. The LFMA spectra for all ribbons were performed at X-band (∼9.5 GHz), at temperatures below Curie temperature. Additionally, for annealed ${\mathrm{Ni}}_{45}{\mathrm{Co}}_{5}{\mathrm{Mn}}_{35.5}{\mathrm{In}}_{14.5}$ ribbons, the LFMA signal dependencies of the external magnetic field modulation amplitude, modulation frequency, microwave power and microwave magnetic field phase were registered. These results confirm the resonant character of LFMA. To determine the basic EMR parameters, such as linewidth and resonance field, the experimental data were fitted by the Dyson function. The LFMA signal is satisfactorily matched by the two lines, and the variability of the component lines with temperature is remarkable.

## 1. Introduction

In some materials, microwave absorption can be tuned by a very low magnetic field, and in that case, this effect is known as low-field microwave absorption (LFMA). This phenomenon was explored in different magnetic materials, such as amorphous ribbons, nanoparticles, nanowires, magnetic thin films, and other soft magnetic materials [1,2,3,4,5,6,7]. However, a certain theoretical or experimental explanation of LFMA is not known. The occurrence of the LFMA signal depends on the magnetic ordering and appears only in the ferromagnetic state, when long-range magnetic interactions dominate. Various explanations can be found in the literature for the zero-field peak in ferromagnetic materials, including some authors pointing to the non-resonant origin of this peak [1,2]. In amorphous ${\mathrm{Co}}_{66}{\mathrm{Fe}}_{4}B_{12}{\mathrm{Si}}_{13}{\mathrm{Nb}}_{4}\mathrm{Cu}$ ribbons, the presence of the LFMA is associated with the absorption of electromagnetic radiation by the spin system, which is associated with the configuration of magnetic domains, and also strongly depends on magnetocrystalline anisotropy. In addition, there is a relationship between this phenomenon and magneto-impedance [1,8]. The studies discussed in these papers show the relationship between the maximum in the magneto-impedance curve and the minimum or maximum of the LFMA signal. Low-field microwave absorption is also observed in superconductors materials, where it is known as non-resonant microwave absorption, and it is used as an indicator of the superconducting state [9,10,11].

In our previous work of the electron magnetic resonance (EMR) of the annealed ${\mathrm{Ni}}_{50}{\mathrm{Mn}}_{35.5}{\mathrm{In}}_{14.5}$ ribbons [12], the LFMA signal was observed for the first time in the Ni-Mn-based family. Furthermore, the influence of the sample geometry on the occurrence of LFMA in ${\mathrm{Ni}}_{47}{\mathrm{Co}}_{3}{\mathrm{Mn}}_{35.5}{\mathrm{In}}_{14.5}$ alloys was examined [13]. Recently, Modak et al. [14] denoted the existence of the LFMA signal in Ni-Mn-Sn and Ni-Mn-In, but the origin of the LFMA signal in this group of materials has not yet been studied in detail. This article discusses the unpublished EMR data of ${\mathrm{Ni}}_{50-x}{\mathrm{Co}}_{x}{\mathrm{Mn}}_{35.5}{\mathrm{In}}_{14.5}$ ribbons and provides an overview of the LFMA that was omitted in our previous studies.

## 2. Materials and Method

In this study, three ribbons ${\mathrm{Ni}}_{50-x}{\mathrm{Co}}_{x}{\mathrm{Mn}}_{35.5}{\mathrm{In}}_{14.5}$ with different contents of Co are investigated. Alloys in ribbon form with composition ${\mathrm{Ni}}_{50-x}{\mathrm{Co}}_{x}{\mathrm{Mn}}_{35.5}{\mathrm{In}}_{14.5}$ (x = 0, 3, 5) were prepared using the melt-spinning method, according to the procedure described in Ref. [15]. The samples are labeled NC0MI-1, NC3MI-1, and NC5MI-1 for 0, 3, and 5 at.% cobalt content, respectively. The second series of samples before measurements was annealed at 1173 K for 30 min and then slowly cooled in a furnace for 12 h. Annealed samples are labeled NC0MI-2, NC3MI-2, and NC5MI-2. The LFMA signal was observed only for samples listed in Table 1.

**Figure 1:**
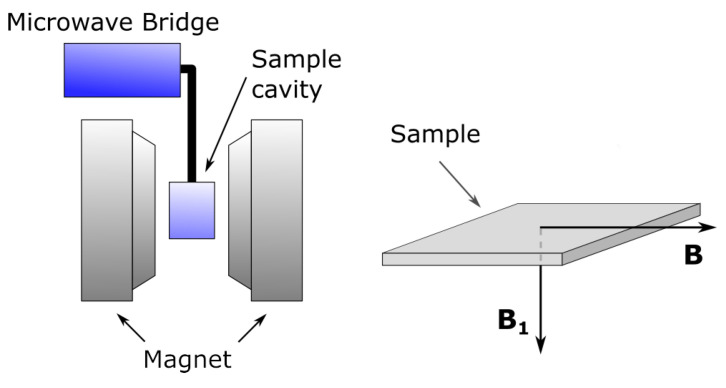
Schematic of the EMR setup, used for detection of LFMA signal and configuration of the sample with respect to the magnetic field vectors. B and $B_{1}$ denote the external magnetic field and microwave magnetic field, respectively.

Electron magnetic resonance (EMR) measurements were performed using the Bruker ELEXYS E580 spectrometer equipped with the Bruker liquid N gas flow cryostat with the 41131 VT digital controller (Bruker Analytische Messtechnik, Rheinstetten, Germany) in the X-band (9.44 GHz). The LFMA spectra were registered using a standard super-high-Q resonator (ER 4123D) at a 100 kHz magnetic field modulation with amplitude of 1 Gs. Figure 1 illustrates the sample position with respect to the magnetic field vectors. During the measurement, the sample is placed into cavity such that the external magnetic field vector is parallel to the ribbon plane (in-plane configuration). The EMR parameters were obtained by fitting the theoretical curve to experimental data and using OriginPro 8.5 software (OriginLab, Northampton, MA, USA). For fitting, the Dyson function was applied [16,17].

## 3. Results

**Figure 2:**
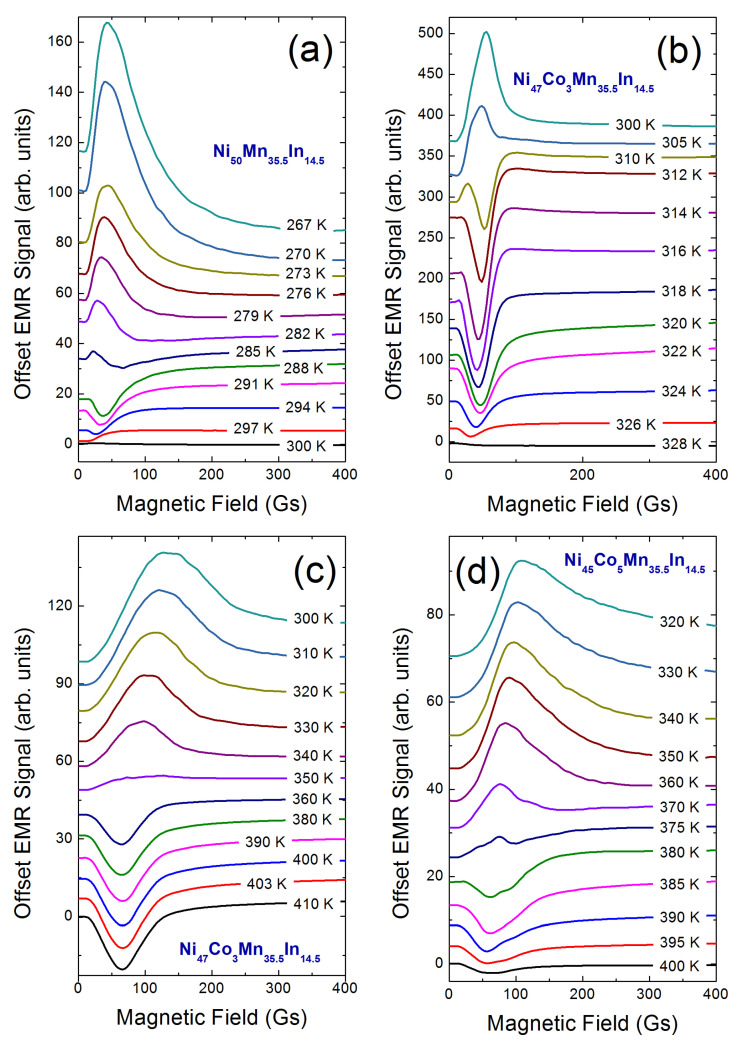
The temperature evolution of LFMA signal for ${\mathrm{Ni}}_{50-x}{\mathrm{Co}}_{x}{\mathrm{Mn}}_{35.5}{\mathrm{In}}_{14.5}$ ribbons: annealed ${\mathrm{Ni}}_{50}{\mathrm{Mn}}_{35.5}{\mathrm{In}}_{14.5}$ (**a**), as-spun ${\mathrm{Ni}}_{47}{\mathrm{Co}}_{3}{\mathrm{Mn}}_{35.5}{\mathrm{In}}_{14.5}$ (**b**), annealed ${\mathrm{Ni}}_{47}{\mathrm{Co}}_{3}{\mathrm{Mn}}_{35.5}{\mathrm{In}}_{14.5}$ (**c**) and annealed ${\mathrm{Ni}}_{45}{\mathrm{Co}}_{5}{\mathrm{Mn}}_{35.5}{\mathrm{In}}_{14.5}$ (**d**).

For the sample ${\mathrm{Ni}}_{50-x}{\mathrm{Co}}_{x}{\mathrm{Mn}}_{35.5}{\mathrm{In}}_{14.5}$, one can observe two signals connected to the absorption of microwave radiation, that is, in the high field (∼330 mT) the typical EMR/FMR signal is related to the spins of ${\mathrm{Mn}}^{2+}$ ion, and in the low-field, the LFMA signal. In these materials, the mainline (EMR line) consists of a single broad Dyson-type line and due to the polycrystalline character of the sample, the dominant contribution to the spin Hamiltonian is the Zeeman term, that is the interaction between an electron spin and an external magnetic field [18]. The character of the EMR line for ${\mathrm{Ni}}_{50-x}{\mathrm{Co}}_{x}{\mathrm{Mn}}_{35.5}{\mathrm{In}}_{14.5}$ ribbons was studied and described in detail in our prior papers [12,13,18,19,20,21]. Figure 2 shows LFMA signals for selected temperatures for the NC0MI-2, NC3MI-1, NC3MI-2 and NC5MI-2 samples, respectively. For soft ferromagnets, the LFMA signal appears below the Curie temperature ($T_{C}$), and therefore Figure 2 shows the spectra recorded in different temperature ranges, depending on $T_{C}$ for this particular sample. The values of $T_{C}$ for each sample are listed in Table 2. The temperature dependencies for each sample have some common characteristics, more precisely, at high temperatures, near the $T_{C}$, the LFMA signal resembles the minimum of a typical EMR line or the EMR signal with overlapped lines that have the phase opposite to the EMR mainline for Ni-Co-Mn-In alloys (see [13]). Furthermore, for each sample, with temperature decreasing, the characteristic temperature $T^{*}$ can be observed, which is equal to 285 K, 310 K, 350 K and 375 K for NC0MI-2, NC3MI-1, NC3MI-2 and NC5MI-2, respectively. For individual samples below $T^{*}$ temperature, the LFMA signal changes phase.

This is especially interesting for the NC3MI-2 sample because the $T^{*}$ temperature can be connected to the formation of magnetic ordering. First, for this sample, the Curie temperature $T_{C}$ determined from the EMR data and the vibrating sample magnetometer (VSM) are very different [18], i.e., the $T_{C}$ determined from the VSM is equal to 364 K, while the physical quantity determined from the EMR result is equal to 421 K. This discrepancy is the consequence of the character of both methods. The EMR technique is sensitive to the configuration of the magnetic ion environment, unlike the VSM technique, which provides information on macro-scale magnetic ordering. The higher Curie temperature (421 K), determined from the temperature dependence of the EMR line, reveals the formation of local ordering, while the $T^{*}$ temperature can be connected with the existence of ferromagnetic ordering in the whole volume of the sample.

Due to the metallic character of the investigated samples and the existence of the skin-depth effect [22,23], which causes the appearance of the dispersion part in the EMR lines recorded for Ni-Co-Mn-In alloys, the main EMR line can be satisfactorily fitted by the Dyson line [12,18] described by formula (1) [17]:

(1) $$\frac{dP}{dB}\propto \frac{d}{dB}\left(\frac{\Delta B+\alpha (B-B_{R})}{{\left(B-B_{R}\right)}^{2}+\Delta B^{2}}+\frac{\Delta B+\alpha (B+B_{R})}{{\left(B+B_{R}\right)}^{2}+\Delta B^{2}}\right),$$

where *B* is the induction of the magnetic field, α denotes the asymmetry parameter describing the proportion of absorption and dispersion part in EMR signal, ΔB is the EMR linewidth, and $B_{R}$ is the resonance field.

**Figure 3:**
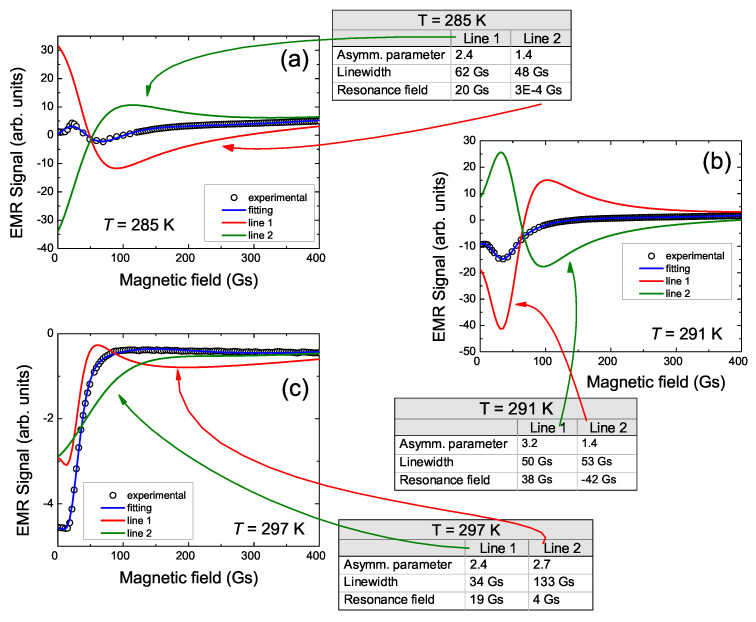
Representative LFMA signals for ${\mathrm{Ni}}_{50}{\mathrm{Mn}}_{35.5}{\mathrm{In}}_{14.5}$ annealed ribbon, their fitting by Dyson curves and fitting parameters at 286 K (**a**), 291 K (**b**) and 297 K (**c**).

**Figure 4:**
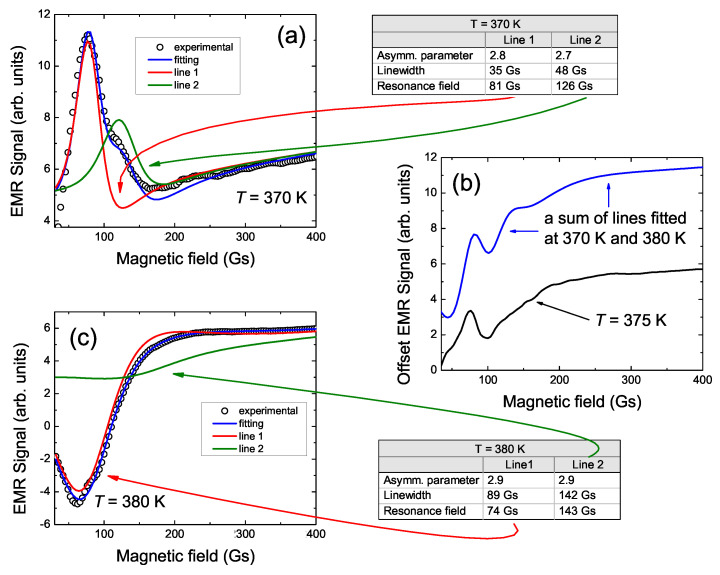
Representative LFMA signals for ${\mathrm{Ni}}_{45}{\mathrm{Co}}_{5}{\mathrm{Mn}}_{35.5}{\mathrm{In}}_{14.5}$ annealed ribbon, their fitting by Dyson curves and fitting parameters at 370 K (**a**), 375 K (**b**) and 380 K (**c**).

In order to characterize the LFMA signal, an attempt was made to fit the experimental LFMA data using the Dyson curve. These attempts indicated that the LFMA signal for all ${\mathrm{Ni}}_{50-x}{\mathrm{Co}}_{x}{\mathrm{Mn}}_{35.5}{\mathrm{In}}_{14.5}$ samples consists of two lines of Dyson shape. Two Dysonian lines observed in the EMR/FMR spectrum [13] additionally confirm the correctness of such a deconvolution of the LFMA signal. A similar correlation between the LFMA and FMR lines is also observed for other materials [1,2,3,4,5]. The example of fitting the experimental LFMA spectra for NC0MI-2 and NC5MI-2 by two Dyson curves is shown in Figure 3 and Figure 4, respectively. At high temperatures, two overlapping lines which consisting of LFMA have an opposite phase to the high-field EMR line. Additionally, with the reduction in temperature, the LFMA changes the phase.

For an annealed ribbon without cobalt (Figure 3), the fittings were performed within the temperature range 279 K ≤T≤ 300 K. At the highest temperatures, the LFMA signal is well fitted by two narrow, asymmetric lines. The first of them labeled as line 1 is very narrow. The second line labeled as line 2 is very narrow too, but is broader than line 1 by about 100 Gs, whereas its resonance field is very close to $B_{R}$ of line 1. At these temperatures, both lines have an opposite phase to the main EMR line. With the reduction in temperature, the parameters of lines 1 and 2 are changed. The intensity and linewidth of both lines are similar, but line 2 changes the phase (see Figure 3b), while line 1 still has an opposite phase to the EMR line [13].

For the NC5MI-2 sample (Figure 4), the fittings were performed within the temperature range 320 K ≤T≤ 400 K. At the highest temperatures, the LFMA signal is well fitted by two lines. The line labeled as line 1 is narrower and has stronger intensity. The line 2 is broader than line 1 about 50 Gs, whereas its intensity is much less than that of line 1. Both lines have an opposite phase to the main EMR line, and they have similar value of the resonance field. Below $T^{*}$ temperature, at 370 K, the LFMA signal, and thereby the two component lines, change phase (see Figure 4a). The proportion between the intensity of line 1 and 2 is conserved, and the intensity of both of them increase significantly, while their linewidth decreases. Figure 4b presents the LFMA signal for NC5MI-2 at 370 K. As one can see, at this temperature, the LFMA line changes phase. What is more is that at this temperature, the fitting of the two lines is no longer satisfactory, while the sum of lines fitted to the experimental data of 370 K and 380 K reproduces this line well. For the problem of fitting at this temperature, and generally for all samples, the observance of the LFMA signal phase changing can be explained by the changes of the resonance condition with temperature. During the measurements, with the temperature evolution, the surrounding magnetic ions in our samples change, which results in the changing of the resonant condition which also is visible in the measured parameter. During the experiment, the settings were adjusted mainly because of the EMR line, which lies in the high-field region of the magnetic field, which may explain the changes in the LFMA signal phase. To sum up, the $T^{*}$ temperature is observed below the Curie temperature because it is correlated, e.g., with magnetic ordering and ferromagnetic interactions. The parameters which describe both component lines evolve with temperature changing and $T^{*}$ is the temperature at which these components balance each other.

**Figure 5:**
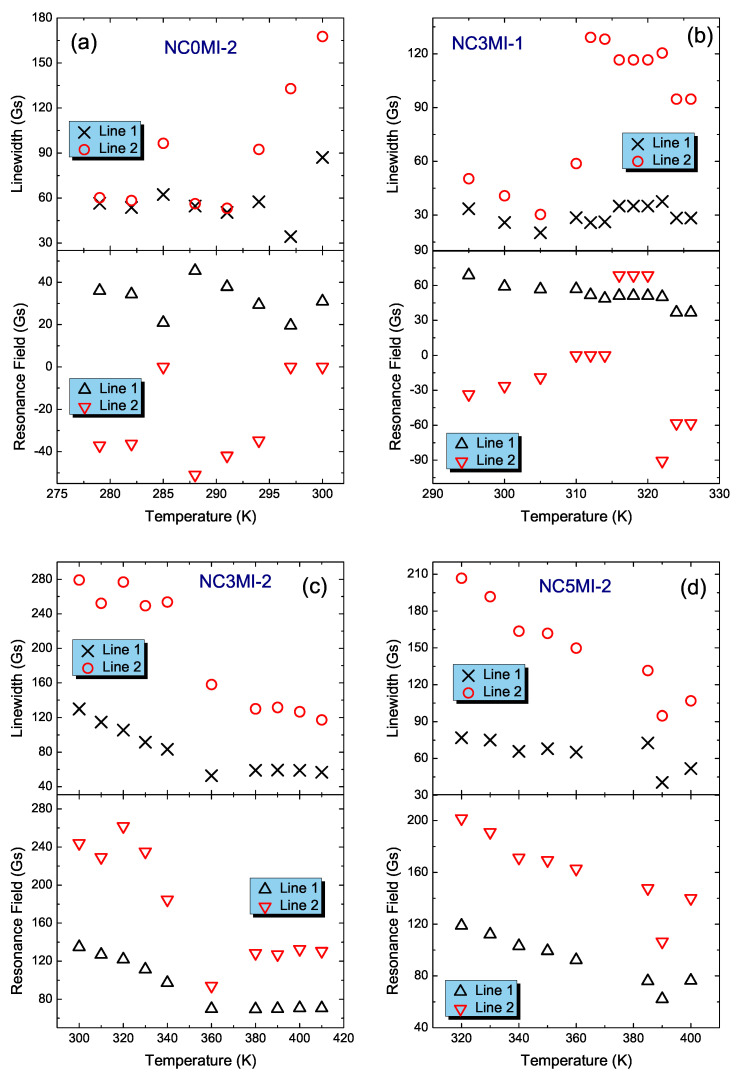
Linewidth and resonance field as a function of the temperature for two fitted lines of in situ ${\mathrm{Ni}}_{47}{\mathrm{Co}}_{3}{\mathrm{Mn}}_{35.5}{\mathrm{In}}_{14.5}$ (**b**) and annealed ribbons: ${\mathrm{Ni}}_{50}{\mathrm{Mn}}_{35.5}{\mathrm{In}}_{14.5}$ (**a**), ${\mathrm{Ni}}_{47}{\mathrm{Co}}_{3}{\mathrm{Mn}}_{35.5}{\mathrm{In}}_{14.5}$ (**c**) and ${\mathrm{Ni}}_{45}{\mathrm{Co}}_{5}{\mathrm{Mn}}_{35.5}{\mathrm{In}}_{14.5}$ (**d**).

Figure 5 shows the temperature dependencies of the linewidth and the resonance field for NC0MI-2, NC3MI-1, NC3MI-2 and NC5MI-2 samples, respectively. For NC0MI-2 and NC3MI-1 samples, the ΔB(T) for line 1 changes slightly in the whole temperature range, e.g., for NC0MI-2, it oscillates about 60 Gs, while for NC3MI-1, it is equal to approx 30 Gs. Similarly, the ΔB of line 1 determined for the NC5MI-2 sample is weakly temperature dependent and below 380 K, does not exceed 75 Gs.

In contrast, for NC3MI-2 (see Figure 5c), ΔB of line 1 in the temperature range 360 K ≤T≤ 410 K oscillates about 60 Gs. With further reduction in temperature, the ΔB of line 1 increases linearly up to 130 Gs. Comparing all these values of linewidth with the linewidth of EMR lines in these materials, one can see that ΔB of LFMA is lesser by an order of magnitude. A similar temperature dependence of ΔB is observed for this sample for the line labeled as line 2; only at the high temperature does the linewidth oscillate about 120 Gs, and then, with the reduction in temperature it increases up to 280 Gs. For NC5MI-2, the linewidth of line 2 increases with temperature decreasing, but this dependency is linear. For other samples, that is, NC0MI-2 and NC3MI-1, the ΔB(T) has an irregular character.

The down panels of Figure 5a,b include the temperature dependence of the resonance field for the NC0MI-2 and NC3MI-1 samples, respectively. For both samples, the $B_{R}\left(T\right)$ for line 1 has a linear character. The same dependencies for line 2 change irregularly and take $B_{R}$ values below zero. The temperature dependencies of the resonance field for the two last samples presented in Figure 5c,d have a similar character to their temperature dependencies of linewidth. The $B_{R}$ of line 1 of the sample NC3MI-2 in the temperature range 360 K ≤T≤ 410 K oscillates about 70 Gs and then with decreasing temperature, the resonance field linearly shifts to 140 Gs. For line 2, the $B_{R}$ in the temperature range 380 K ≤T≤ 410 K is approximately 140 Gs, and with decreasing temperatures, the $B_{R}$ value starts increasing. The temperature dependence for both lines in the NC5MI-2 sample is the most regular. The resonance field increases linearly with reduction in the temperature up to 120 Gs and 200 Gs for line 1 and line 2, respectively.

The standard X-band EMR spectra are registered between 0 and 7000 Gs, and therefore, in this regime of a magnetic field, the EMR/FMR line is expected. However, the location of the EMR signal depends not only on the material from which the sample is made, but also on other quantities or experiment settings, such as temperature and microwave (MW) frequency, e.g., for Ni-Mn-In ribbons, in the paramagnetic state, the resonance field is ∼3300 Gs. In our experiment, one also scanned the NC5MI-2 sample from the negative field value −3000 Gs to 1 T, so that one could see the entire LFMA line.

**Figure 6:**
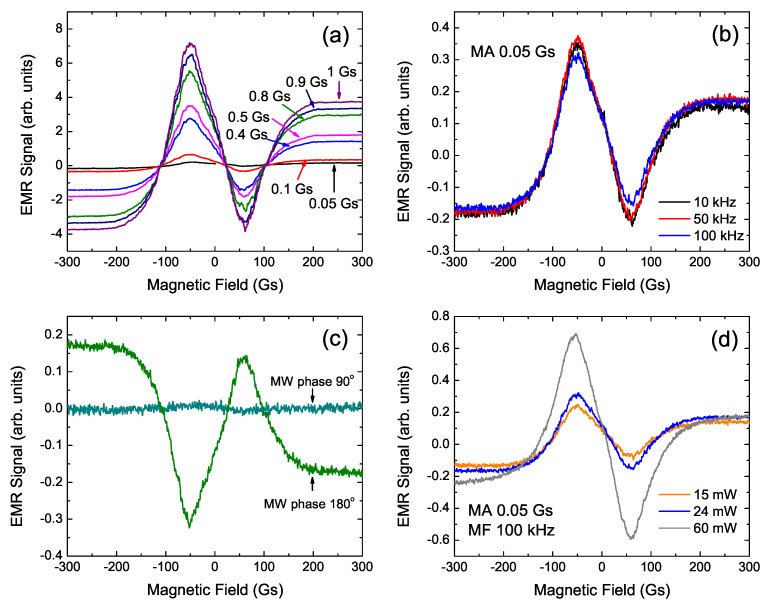
The LFMA signal for ${\mathrm{Ni}}_{45}{\mathrm{Co}}_{5}{\mathrm{Mn}}_{35.5}{\mathrm{In}}_{14.5}$ ribbon at 370 K: signal changes depending on modulation amplitude at modulation frequency 100 kHz (**a**); the LFMA signal registered for several modulation frequencies (modulation amplitude equal to 0.05 Gs) (**b**); the LFMA signal obtained in the two different microwave radiation phase (**c**) and microwave power dependence of the LFMA signal measured at modulation frequency 100 kHz and modulation amplitude equal to 0.05 Gs (**d**).

In order to confirm the resonance character of low-field lines observed in the EMR spectra for Ni-Co-Mn-In ribbons, one performed the EMR measurements with various instrument settings [24]. Figure 6 shows the LFMA signal dependence of basic electron magnetic resonance parameters, e.g., external magnetic field modulation amplitude (MA), modulation frequency (MF), microwave phase and microwave power. One can see that the LFMA signal is dependent on these all parameters. The experiment set related to external magnetic field, such as modulation amplitude and modulation frequency values changes the intensity of LFMA. With the reduction in modulation frequency value, the intensity of LFMA slightly decreases, while the LFMA increases drastically with modulation amplitude increasing (MF fixed at 100 kHz). Similar behavior was observed in the LFMA in ${\mathrm{Zn}}_{1-x}\left(\mathrm{Mn}:\mathrm{Fe}{\left(\mathrm{Ni}\right)}_{x}\right)O$ [25].

Figure 6d shows the variation of LFMA with microwave power for the annealed ${\mathrm{Ni}}_{45}{\mathrm{Co}}_{5}{\mathrm{Mn}}_{35.5}{\mathrm{In}}_{14.5}$ ribbon at 370 K. Here, one can see a strong relationship between microwave power and LFMA intensity, that is, with increasing the microwave power, the intensity increases significantly. Gavi et al. [26] observed the same phenomenon in a thin layer of FeSi and explained this observation by the change in the magnetic field strength in the cavity of the spectrometer caused by the change in microwave power. Increasing the intensity of the magnetic field inside the cavity increases the microwave current in the metallic sample; due to the existence of the skin depth effect, one can talk about changes of that current value on the sample surface. What is more, the increasing of the microwave current leads to an increased microwave power loss in the sample [25,26], which, due to impedance, is ohmic. Montiel et al. [8] reported that this phenomenon indicates a common origin of the LFMA signal and magneto-impedance.

Furthermore, comparing the LFMA spectra for Figure 6 with a line for Figure 4d, one can see that the line has an opposite phase. The explanation for this is that the EMR and LFMA signals for this alloy show a temperature hysteresis, and therefore the signal recorded at the same temperature during cooling and heating differs in LFMA signal phase. Therefore, for measurements, while the sample is cooled (Figure 6), the temperature $T^{*}$, at which the phase change of the LFMA signal occurs, will be lower than for measurements carried out while the sample is heated (Figure 4). In our previous paper, a similar hysteresis was observed for the temperature dependency of the EMR signal for the ${\mathrm{Ni}}_{45}{\mathrm{Co}}_{5}{\mathrm{Mn}}_{35.5}{\mathrm{In}}_{14.5}$ as-cast ribbon [20].

The last dependency presented in Figure 6c concerns the microwave radiation phase, i.e., the classical parameter of electron magnetic spectroscopy. As one can see, the change in the microwave phase of 90 deg causes the LFMA signal to disappear, while for a phase equal to 180 deg, there is only a LFMA phase change [27]. This behavior is the next confirmation of the resonant character of LFMA in Ni-Co-Mn-In.

## 4. Conclusions

The LFMA signal was detected in all samples listed in Table 1. Three of them were annealed at 1173 K, the last one labeled by NC3MI-2 was not. Careful investigation using various instrumental settings, i.e., the magnetic field modulation amplitude, modulation frequency, change in phase and power of microwave confirmed that the low-field lines exhibit resonance character.

The LFMA lines were well fitted by the Dysonian formula (1), which take into account the skin effect characteristic for the metallic samples. The fitting procedure revealed that each of the investigated lines is decomposed onto two lines, each of them being the Dyson line. Usually, the LFMA line appears together with the EMR/FMR line (see ref. [12,13,18]), and if the FMR line has two components, the LFMA should also have two components. The parameters of the component lines were determined and, additionally, their changes with temperature were plotted. The temperature dependence of resonance field and linewidth of those components are similar in samples NC3MI-2 and NC5MI-2, their values decreasing with increasing temperature. For the other two samples NC0MI-2 and NC3MI-1, the characteristic changes of these parameters are not so regular.

For all investigated samples, there is a characteristic temperature $T^{*}$ in which the phase of LFMA signals is changed to the opposite one. The lowest value of this temperature is observed in the NC0MI-2 sample, and the highest, for the annealed NC5MI-2 ribbon. The value of these parameters varies with the composition of the sample and the thermal treatment.

Changes in temperature $T^{*}$ coincides with the shift in Curie temperatures that can be assigned to the investigated samples. This is a symptom of the more general dependence of the characteristic temperatures of Heusler alloys. It is observed that the temperature associated with structural changes increases with annealing and with an increase in the cobalt content in such structures.

## Figures and Tables

**Table 1:** Investigated samples.

Label	Composition	Annealing
NC0MI-2	${\mathrm{Ni}}_{50}{\mathrm{Mn}}_{35.5}{\mathrm{In}}_{14.5}$	Yes
NC3MI-1	${\mathrm{Ni}}_{47}{\mathrm{Co}}_{3}{\mathrm{Mn}}_{35.5}{\mathrm{In}}_{14.5}$	No
NC3MI-2	${\mathrm{Ni}}_{47}{\mathrm{Co}}_{3}{\mathrm{Mn}}_{35.5}{\mathrm{In}}_{14.5}$	Yes
NC5MI-2	${\mathrm{Ni}}_{45}{\mathrm{Co}}_{5}{\mathrm{Mn}}_{35.5}{\mathrm{In}}_{14.5}$	Yes

**Table 2:** The Curie temperature determined from temperature dependency of EMR integral intensity for ${\mathrm{Ni}}_{50-x}{\mathrm{Co}}_{x}{\mathrm{Mn}}_{35.5}{\mathrm{In}}_{14.5}$ and $T^{*}$ temperature. The $T_{C}$ values were taken from the literature [13].

Label	Composition	$T_{C}$ (K)	$T^{*}$ (K)
NC0MI-2	${\mathrm{Ni}}_{50}{\mathrm{Mn}}_{35.5}{\mathrm{In}}_{14.5}$	300	285
NC3MI-1	${\mathrm{Ni}}_{47}{\mathrm{Co}}_{3}{\mathrm{Mn}}_{35.5}{\mathrm{In}}_{14.5}$	340	310
NC3MI-2	${\mathrm{Ni}}_{47}{\mathrm{Co}}_{3}{\mathrm{Mn}}_{35.5}{\mathrm{In}}_{14.5}$	421	350
NC5MI-2	${\mathrm{Ni}}_{45}{\mathrm{Co}}_{5}{\mathrm{Mn}}_{35.5}{\mathrm{In}}_{14.5}$	414	375

## Data Availability

Not applicable.
